# The SUV39H1 inhibitor chaetocin induces differentiation and shows synergistic cytotoxicity with other epigenetic drugs in acute myeloid leukemia cells

**DOI:** 10.1038/bcj.2015.37

**Published:** 2015-05-15

**Authors:** Y-S Lai, J-Y Chen, H-J Tsai, T-Y Chen, W-C Hung

**Affiliations:** 1 National Institute of Cancer Research, National Health Research Institutes, Tainan, Taiwan; 2 Division of Hematology/Oncology, Department of Internal Medicine, National Cheng Kung University Hospital, Tainan, Taiwan; 3 Institute of Basic Medical Sciences, College of Medicine, National Cheng Kung University, Tainan, Taiwan

## Abstract

Epigenetic modifying enzymes have a crucial role in the pathogenesis of acute myeloid leukemia (AML). Methylation of lysine 9 on histone H3 by the methyltransferase G9a and SUV39H1 is associated with inhibition of tumor suppressor genes. We studied the effect of G9a and SUV39H1 inhibitors on viability and differentiation of AML cells and tested the cytotoxicity induced by combination of G9a and SUV39H1 inhibitors and various epigenetic drugs. The SUV39H1 inhibitor (chaetocin) and the G9a inhibitor (UNC0638) caused cell death in AML cells at high concentrations. However, only chaetocin-induced CD11b expression and differentiation of AML cells at non-cytotoxic concentration. HL-60 and KG-1a cells were more sensitive to chaetocin than U937 cells. Long-term incubation of chaetocin led to downregulation of SUV39H1 and reduction of H3K9 tri-methylation in HL-60 and KG-1a cells. Combination of chaetocin with suberoylanilide hydroxamic acid (SAHA, a histone deacetylase inhibitor) or JQ (a BET (bromodomain extra terminal) bromodomain inhibitor) showed synergistic cytotoxicity. Conversely, no synergism was found by combining chaetocin and UNC0638. More importantly, chaetocin-induced differentiation and combined cytotoxicity were also found in the primary cells of AML patients. Collectively, the SUV39H1 inhibitor chaetocin alone or in combination with other epigenetic drugs may be effective for the treatment of AML.

## Introduction

Epigenetic alterations contribute to the pathogenesis of hematopoietic malignancies including acute myeloid leukemia (AML). Aberrant promoter methylation inactivates the expression of tumor suppressor genes which leads to blockage of differentiation and deregulated proliferation.^1, 2^ Targeting the epigenetic modifying enzymes like DNA methyltransferases, histone methyltransferases or histone deacetylase (HDAC) becomes an important area for the development of anti-cancer drugs.^3, 4^ Currently, several epigenetic drugs have been approved for cancer treatment. For example, the HDAC inhibitors SAHA (also known as Vorinostat) and Romidepsin (Istodax) are used for the treatment of cutaneous T-cell lymphoma. The DNA methyltransferase inhibitors 5′-azacitidine (Vidaza) and decitabine (Dacogen) are therapeutic drugs for myelodysplastic syndrome.

Epigenetic regulation includes DNA methylation and histone modification, two biological processes which are strongly associated. Previous studies demonstrated that methylation of lysine 9 on histone H3 (H3K9) generates a ‘silence code' which is critical for the heterochromatin assembly and is sufficient for initiation of gene repression.^5, 6, 7^ In addition, a close coupling between H3K9 methylation and DNA methylation has been found in the transcription of a number of target genes.^8^ In mammalian cells, mono- and di-methylation of H3K9 is mainly mediated by the lysine methyltransferase G9a and its related molecule G9a-like protein (GLP) which exists predominantly as a G9a/GLP complex.^9^ A number of biological functions of G9a/GLP including germ cell development, pluripotency, immune regulation and cell proliferation have been suggested.^10^ Upregulation of G9a is found in many solid tumors such as breast cancer, lung cancer, colon cancer and prostate cancer.^11, 12, 13^ An oncogenic role of this methyltransferase in AML has also been suggested recently.^14^ A small molecular inhibitor of G9a, BIX-01294, was firstly reported by KubiceK *et al.*^15^ and this compound reduced H3K9 di-methylation and reactivated the expression of several G9a target genes in cell-based assays. Another inhibitor UNC0638 with higher selectivity was developed and showed anti-cancer activity on MCF-7 breast cancer cells.^16^

After mono- and di-methylation, H3K9 is further tri-methylated by SUV39H1. Tri-methylated H3K9 generated a binding site for the HP1 protein, a heterochromatic adaptor molecule implicated in both gene silencing and supra-nucleosomal chromatin structure, which led to inhibition of gene transcription.^17^ The possible involvement of SUV39H1 in leukemia was suggested by the findings that SUV39H1 is an associated protein of the transcription factor AML1 (also known as RUNX1) which has an important role in the regulation of proliferation and self-renewal of hematopoietic stem cells.^18, 19^ The interaction between AML1 and SUV39H1 and G9a is required for transcriptional repression and bone marrow immortalization.^20^ Greiner *et al.*^21^ reported that chaetocin, a fungal mycotoxin belongs to the class of 3–6 epidithio-diketopiperazines originally isolated from *Chaetomium minutum* is a specific inhibitor of SUV39H1. However, subsequent evidence suggested chaetocin is a nonspecific inhibitor of histone methyltransferases and might also inhibit G9a activity in addition to SUV39H1 at higher concentration.^22^

Because the alteration of H3K9 methylation is generally found in AML cells and is associated with blockage of differentiation and deregulated proliferation, we tested the differentiation-inducing and cytotoxic effect of G9a and SUV39H1 inhibitor in AML cell lines and primary AML cells. In addition, we studied the effect of these inhibitors in combination with a HDAC inhibitor and a newly developed BET (bromodomain extra terminal protein) bromodomain inhibitor which bind competitively to acetyl-lysine recognition motifs to suppress the interaction between BET proteins and acetylated histone markers.

## Materials and methods

### Cell culture

Human AML cell lines were purchased from the Bioresource Collection and Research Center (Hsin-Zhu, Taiwan). KG-1a cells were cultured in IMDM (Iscove's modified Dulbecco's medium) medium containing 15% fetal bovine serum. HL-60 and U937 cells were maintained in RPMI (Roswell Park Memorial Institute medium) medium containing 10% fetal bovine serum.

### Material

Chaetocin were purchased from Cayman Chemical Company (Ann Arbor, MI, USA). ASK Liu's stain reagent was purchased from Tonyar Biotech. Inc. (Tao Yuan, Taiwan). Anti-H3K9me2, anti-H3K9me3, anti-H3K27me2 and anti-H3K27me3 antibodies were obtained from Cell Signaling Technology Inc. (Danvers, MA, USA).

### Clinical AML cell samples

Primary leukemia cells were obtained from the bone marrow samples of 11 AML patients with informed consent. This study was approved by the Institutional Review Board of National Cheng Kung University Hospital. The AML cells were purified from bone marrow using Ficoll density-gradient centrifugation and stored in liquid nitrogen. Cells were recovered and cultured in RPMI medium containing 10% fetal bovine serum for drug testing.

### Cytotoxicity assay

AML cell lines and patient's AML cells were treated with different concentrations of chaetocin in combination with SAHA or JQ-1. After 24 h, cells were collected by centrifugation and stained with trypan blue reagent. Viable cells were counted under a light microscope. The concentration causing 50% of growth inhibition (IC50) for UNC0638 and chaetocin was also determined.

### Liu's stain and morphological evaluation

Cells were treated with chaetocin for 12 h and then attached on slides using cytospin apparatus (CytoSpin, Thermo Fisher Scientific, Waltham, MA, USA). Cells were stained with Liu's stain reagent A for 1 min and then stained with reagent B for 2.5 min. Cell morphology was observed microscopically.

### RNA extraction and quantitative reverse transcription-PCR analysis

Total RNA was isolated from cells using an RNA extraction kit (Geneaid), and 1 μg of RNA was reverse transcripted to cDNA. Target mRNAs were quantified using real-time PCR reactions with SYBR green fluorescein and actin was served as an internal control. cDNA synthesis was performed at 95 °C for 5 min, and the conditions for PCR were 30 cycles of denaturation (95 °C/45 s), annealing (60 °C/45 s) and extension (72 °C/45 s). The primers used are CD11b forward: 5′-ATATCAGCACATCGGCCTGG-3′ CD11b reverse: 5′-GAACAGCATCACACTGCCAC-3′ Suv39H1 forward: 5′-GGCAACATCTCCCACTTTGT-3′ Suv39H1 reverse: 5′-CAA TACGGACCCGCTTCTTA-3′ G9a forward: 5′-TGGGAAAGGTGACCTCAGAT-3′ G9a reverse: 5′-TCCCTGACTCCTCATCTTCC-3′ actin forward: 5′-TGTTACCAAC TGGGACGACA-3′ actin reverse: 5′-GGGGTGTTGAAGGTCTCAAA-3′.

### Histone extraction and immunoblot analysis

For the extraction of histone proteins, cells were collected by centrifugation and lysed with urea buffer (8 M Urea, 0.1 m NaH_2_PO_4_, 0,01 M Tris-Base, pH 8.0) at room temperature for 30 min on a rotating wheel. Protein quantification was performed using Bio-Rad protein determination reagent. For the detection of histone modifications, 40 μg of total protein extracts were subjected to 15% SDS-polyacrylamide gel electrophoresis separation, and proteins were transferred to polyvinylidene fluoride membranes. Finally, the blots were probed with antibodies against histone H3 or methylated histone H3 and developed by enhanced chemiluminescence reagent.

### Statistical analysis

Statistical analysis was performed by using Student's *t*-test. A *P*-value <0.05 was considered statistically significant.

## Results

### Chaetocin exhibits more potent cytotoxicity than UNC0368 in AML cells

**Figure 1:**
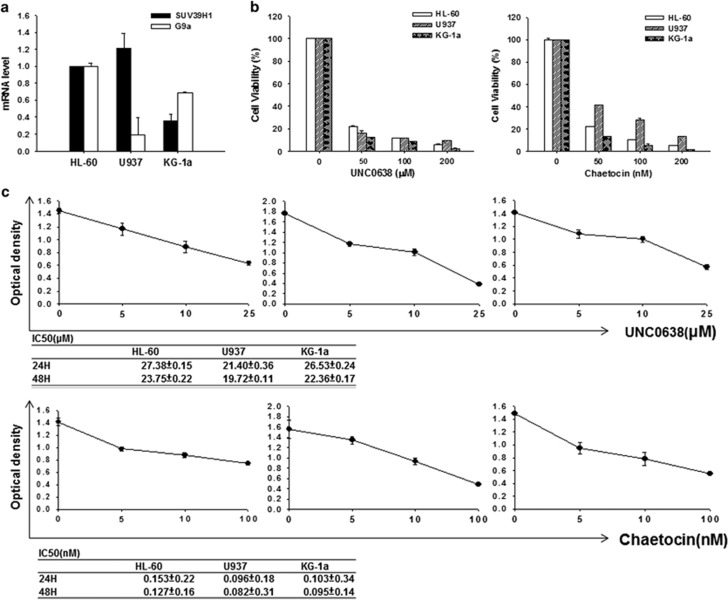
The effect of chaetocin and UNC0638 on human AML cell lines. (**a**) Expression of G9a and SUV39H1 in HL-60, U937 and KG-1a cells. The expression levels of HL-60 were defined as 1. (**b**) Cells were treated with high concentrations of UNC0638 (50–200 μm) or chaetocin (50–200 nm) for 24 h and cell viability was studied by MTT assay. (**c**) Cells were incubated with low concentrations of UNC0638 (0–25 μm) or chaetocin (0–100 nm) for 24 h. IC_50_ values were determined for each cell line.

We examined the expression of G9a and SUV39H1 in three AML cell lines. Among them, HL-60 and U937 cells expressed similar level of SUV39H1 while KG-1a had the lowest expression (Figure 1a). The expression level of G9a was HL-60>KG-1a>U937. We first used high concentration of chaetocin and UNC0368 to treat cells and found that chaetocin at the concentration >50 nm or UNC0638 at the concentration >50 μm induced extensive cell death (Figure 1b). The IC50 of chaetocin calculated at 24 or 48 h after drug incubation was around 82–153 nm for the three cell lines and the IC50 of UNC0638 was around 20 μm (Figure 1c). These data suggested that chaetocin and UNC0638 showed cytotoxicity in AML cells at high concentration and chaetocin is more potent than UNC0638 in cell killing.

### Chaetocin but not UNC0638 induces CD11b expression and differentiation in AML cells

**Figure 2:**
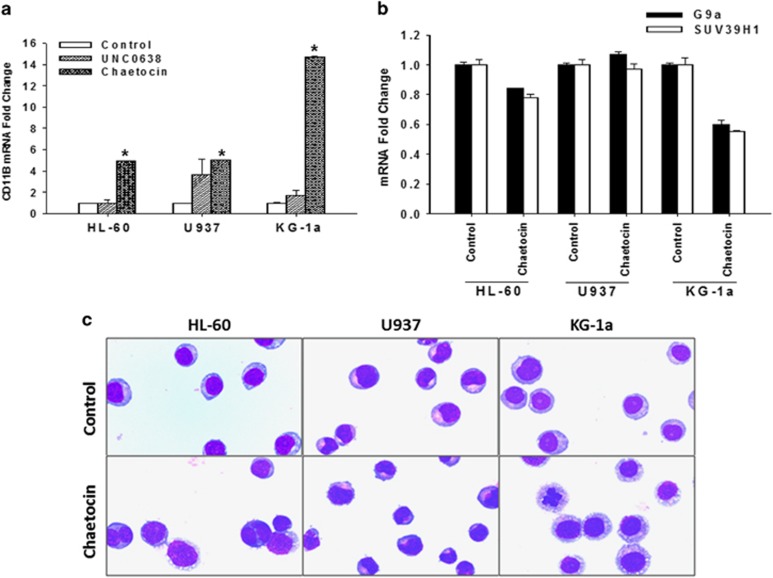
Induction of CD11b expression and differentiation by chaetocin. (**a**) Cells were treated with UNC0638 (20 μm) or chaetocin (20 nm) for 48 h. Expression of CD11b was assayed by real-time RT-PCR. Results represent the mean±s.e. of three independent experiments. *P<0.05 when compared with the control group. (**b**) Cells were treated with or without chaetocin (20 nm) for 48 h and expression of G9a and SUV39H1 was studied by real-time RT-PCR. Results represent the mean±s.e. of three independent experiments. *P<0.05 when compared with the control group. (**c**) Morphology of control or chaetocin-treated cells. Cells were treated with or without chaetocin (20 nm) and collected by cytospin preparations. Cells were stained with Liu's stain and observed under a light microscope.

We next addressed the non-cytotoxic effect of chaetocin and UNC0368. Chaetocin at 20 nm significantly upregulated the expression of CD11b, a myeloid differentiation marker in all three AML cell lines whereas UNC0368 did not have significant effect (Figure 2a). Interestingly, treatment of 20 nm of chaetocin for 48 h reduced SUV39H1 and G9a in HL-60 and KG-1a cells but not in U937 cells (Figure 2b). Morphological examination demonstrated a reduction of nucleus to cytoplasm ration and the appearance of nuclear segmentation in chaetocin-treated HL-60 and KG-1a cells suggesting the induction of differentiation (Figure 2c). Conversely, the morphological change was not obvious in U937 cells.

### Chaetocin reduced H3K9 and H3K27 methylation in HL-60 and KG-1a cells

**Figure 3:**
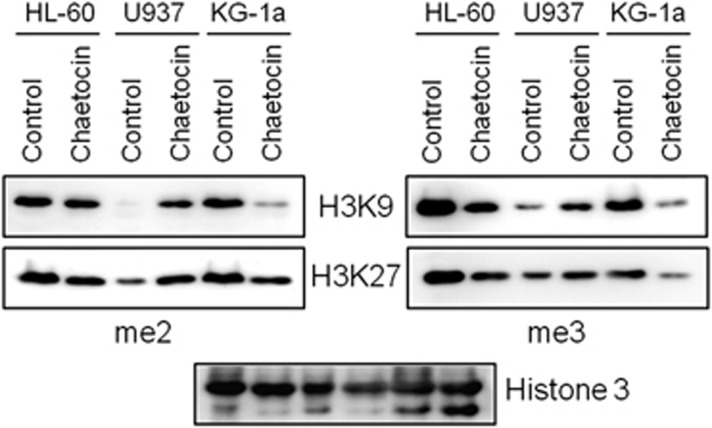
Effect of chaetocin on H3K9 and H3K27 methylation. Cells were treated with or without chaetocin (20 nm) for 48 h and histone proteins were extracted as described in Materials and methods. Methylation status of two repressive markers H3K9 and H3K27 was investigated by immunoblotting.

The effect of chaetocin on H3K9 methylation was investigated. As shown in Figure 3, H3K9 tri-methylation was significantly reduced in HL-60 and KG-1a cells after incubation of 20 nm of chaetocin for 48 h whereas it was increased in U937 cells. An 80% and 20% of reduction of di-methylation of H3K9 was also found in KG-1a and HL-60 cells, respectively. However, chaetocin increased H3K9 di-methylation in U937 cells. Interestingly, chaetocin reduced tri-methylation of another transcription repressive marker H3K27 in KG-1a and HL-60 cells but not in U937 cells. Di-methylation of H3K27 was marginally reduced by chaetocin in KG-1a and HL-60 cells whereas it was increased in U937 cells.

### Chaetocin showed synergistic cytotoxic effect in combination with SAHA and JQ-1

**Figure 4:**
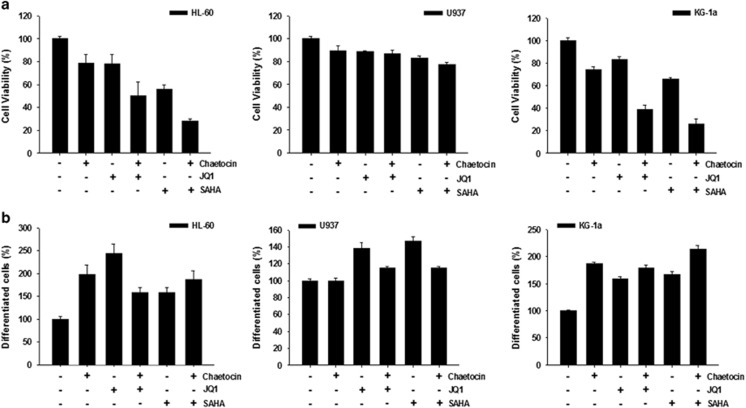
Synergistic cytotoxic effects between chaetocin and two epigenetic inhibitors. (**a**) Cells were incubated in the medium containing different combinations of chaetocin (20 nm), JQ-1 (50 nm) or SAHA (50 μm) for 24 h. Cell viability was studied by MTT assay. (**b**) Cells were treated with different epigenetic inhibitors and collected by cytospin preparations. Cells were stained with Liu's stain and differentiated cells were counted under a light microscope. Results represent the mean±s.e. of three independent experiments.

We next tested the effect of chaetocin in combination with other epigenetic drugs. Chaetocin at 20 nm or JQ-1, a first-in-class small molecule inhibitor of bromodomain and BET proteins^23^ at 50 nm showed minor growth-inhibitory and non-cytotoxic effect in the HL-60 and KG-1a cells (Figure 4a). Combination of chaetocin with JQ-1 enhanced the cytotoxicity in HL-60 and KG-1a cells but not in U937 cells. SAHA at 0.5 μm inhibited 40% of growth of HL-60 and KG-1a cells and reduced the viability to 20–25% when combined with chaetocin (Figure 4a). Morphological examination indicated that JQ-1 and SAHA induced differentiation in all three AML cell lines (Figure 4b). However, no synergy was found when they were combined with chaetocin.

### Differentiation-inducing effect of chaetocin in patient's AML cells

The effect of chaetocin on primary AML cells of 11 patients was tested. The FAB (French–American–British) classification and the percentage of blasts were shown in Table 1. Chetocin increased CD11b expression in 72.7% (8/11) of the AML cells and the typical picture of differentiation of AML cells of one patient (No. 11) was shown in Supplementary Figure 1.

### Cytotoxicity of chaetocin in combination with SAHA and JQ-1 in primary AML cells

Due to the limited cell number, co-treatment study was done in the AML cells of seven patients. Our data demonstrated that combination of chaetocin with SAHA or JQ-1 enhanced cytotoxicity in all of the samples (Table 2).

## Discussion

Three previous studies investigated the effect of chaetocin in AML cells.^24, 25, 26^ However, these studies usually used high concentration (>100 nm) of chaetocin to treat cells to investigate the cell-killing activity. Two important issues for the potential application of chaetocin in cancer therapy should be considered. First, no pharmacokinetics study of chaetocin has been reported. Therefore, whether the concentration used in previous studies could be achievable in serum is unclear and whether cancer patients could tolerate such dose is also unknown. Second, high concentration may mask some specific anti-cancer actions of chaetocin due to the study of a mixture of viable and dead AML cells after drug incubation. In this study, we provide the first evidence that chaetocin could upregulate the expression of CD11b and induce differentiation of AML cell lines and the primary cells of AML patients at low and non-toxic concentration. Our results suggest that chaetocin may be developed as a differentiation-inducing agent similar to the all-trans retinoic acid in acute promyelocytic leukemia. However, we also found that although chaetocin upregulated the expression of CD11b in U937 cells, cell differentiation was not observed. In addition, AML cells from three patients are resistant to chaetocin and no alteration of CD11b expression and morphological change are found (Table 1). It will be an important issue to address the underlying mechanism.

The time- and concentration-dependent action of chaetocin should be noticed. A previous study demonstrated that short-term treatment (8 and 16 h) of chaetocin did not alter global H3K9 tri-methylation.^27^ We also found similar results (data not shown). However, long-term treatment of chaetocin indeed reduced the methylation of H3K9 and H3K27 (Figure 3) indicating this drug indeed changes histone methylation status *in vivo*. Two recent studies demonstrated that chaetocin in addition to inhibit SUV39H1 enzymatic activity also reduced protein level of this methyltransferase.^28, 29^ One of the studies revealed that chaetocin at high concentration acts as a heat shock protein 90 (HSP90) inhibitor and induces degradation of a number of HSP90 client proteins including SUV39H1.^29^ These results make the story of chaetocin more complex and suggest that anti-cancer action of chaetocin is time, dose and cell-context dependent.

We also demonstrated the synergistic cytotoxicity by combining chaetocin and two epigenetic drugs (SAHA and JQ-1). Improved therapeutic effect against leukemia by a combination of chaetocin and a HDAC inhibitor trichostain A was reported previously.^30^ However, trichostain A is not considered as a therapeutic drug due to high toxicity. In the present study, we tested SAHA, an approved drug for cutaneous T-cell lymphoma and demonstrated that combination of chaetocin and SAHA enhanced cytotoxicity to AML cells. Interestingly, we found that a novel class of epigenetic inhibitor JQ-1 also showed synergistic cytotoxicity with chaetocin. BET proteins recognize acetyl-lysine residues presented at different positions of histones proteins and have an important role in the regulation of gene transcription. JQ-1 is the first small molecule inhibitor developed to target BET proteins with the highest binding affinity to BRD4. Three recent studies demonstrated that JQ-1 synergistically kills AML cells when combining with HDAC inhibitors, chemotherapeutic drugs and FLT3 tyrosine kinase inhibitors.^31^ We provide the first evidence showing the combination of chaetocin and JQ-1 enhances cytotoxicity. Inhibition of BRD4 by JQ-1 reduces MYC transcription and attenuates the recruitment of the transcription factor pTEFb.^32, 33^ Whether chaetocin works synergistically with JQ-1 via suppression of MYC and pTEFb warrants future investigation.

Taken together, we conclude that chaetocin induces differentiation of AML cells and shows synergistic cytotoxicity when combines with other epigenetic drugs.

## Figures and Tables

**Table 1:** Effect of chaetocin on CD11b expression of the primary leukemia cells of AML patients

	*Blast %*	*FAB classification*	*CD11B (chaetocin/control)*
AML-1	76	M1	1.46
AML-2	52	M4	5.15
AML-3	68.5	M2	5.51
AML-4	96.5	M1	0.9
AML-5	70	M4	2.48
AML-6	79	M5	1.15
AML-7	92.5	M2	0.5
AML-8	72.5	M1	0.35
AML-9	56	M0	5.97
AML-10	75	M1	2.23
AML-11	50	M5a	1.89

**Table 2:** Cytotoxicity of chaetocin, JQ-1 and SAHA on the primary leukemia cells of AML patients (%)

	*Control*	*CHT*	*JQ-1*	*JQ-1+CHT*	*SAHA*	*SAHA+CHT*
AML-1	100	78	—	—	—	—
AML-2	100	68	—	—	—	—
AML-3	100	50	70	20	60	20
AML-4	100	53	67	33	60	33
AML-5	—	—	—	—	—	—
AML-6	—	—	—	—	—	—
AML-7	100	74	100	59	97	62
AML-8	100	79	67	50	56	42
AML-9	100	14	78	19	46	29
AML-10	100	65	92	46	83	43
AML-11	100	86	81	61	69	63

Abbreviations: CHT, chaetocin; SAHA, suberoylanilide hydroxamic acid.
