# Supplementary figures and images for: The SUV39H1 inhibitor chaetocin induces differentiation and shows synergistic cytotoxicity with other epigenetic drugs in acute myeloid leukemia cells
Source: Blood Cancer J. 2015 May 15;5(5):e313–. doi: 10.1038/bcj.2015.37 (PMC4476016; doi:10.1038/bcj.2015.37)

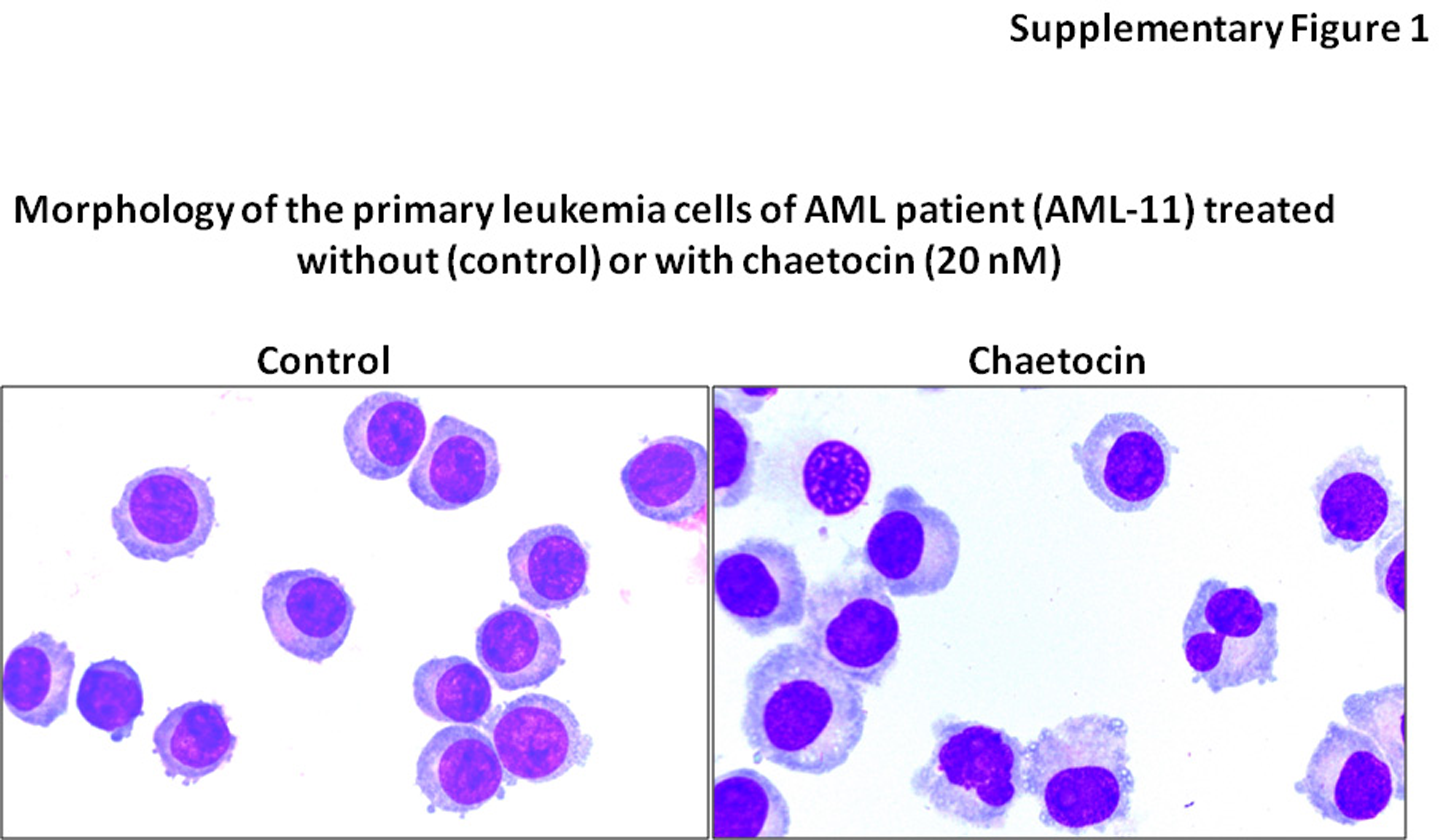

Supplement: Supplementary Figure 1 [file bcj201537x1.tif]
